# Comparable Efficacy and Safety of Teriflunomide versus Dimethyl Fumarate for the Treatment of Relapsing-Remitting Multiple Sclerosis

**DOI:** 10.1155/2021/6679197

**Published:** 2021-07-15

**Authors:** Nasim Nehzat, Omid Mirmosayyeb, Mahdi Barzegar, Reza Vosoughi, Erfane Fazeli, Vahid Shaygannejad

**Affiliations:** ^1^Isfahan Neurosciences Research Center, Isfahan University of Medical Sciences, Isfahan, Iran; ^2^Universal Council of Epidemiology (UCE), Universal Scientific Education and Research Network (USERN), Tehran University of Medical Sciences, Tehran, Iran; ^3^Department of Neurology, School of Medicine, Isfahan University of Medical Sciences, Isfahan, Iran; ^4^University of Toronto, St. Michael's Hospital Multiple Sclerosis Clinic, Toronto, Ontario, Canada

## Abstract

**Background:**

The aim of this observational study is to investigate the efficacy and safety of two approved oral disease-modifying therapies (DMTs) in patients with remitting-relapsing multiple sclerosis (RRMS): dimethyl fumarate (DMF) vs. teriflunomide (TRF).

**Methods:**

A total of 159 RRMS patients (82 on TRF and 77 on DMF) were included. The expanded disability status scale (EDSS), confirmed disability improvement (CDI), confirmed disability progression (CDP), and annualized relapse rate (ARR) were evaluated for the two-year period prior to enrollment in our study. The drug-associated adverse effects (AEs) were recorded. We conducted propensity matching score to compare the efficacy between TRF and DMF.

**Results:**

After matching for the confounders, TRF- and DMF-treated groups were not different in terms of EDSS (*P* value = 0.54), CDI (*P* value = 0.80), CDP (*P* value = 0.39), and ARR (*P* value >0.05). TRF discontinuation occurred in 2 patients (2.43%) due to mediastinitis and liver dysfunction, while a patient (1.29%) discontinued DMF due to depression. Incidence rate of AEs in the TRF-treated group was 81.4%: hair thinning (hair loss) (62.9%), nail loss (20.9%), and elevated aminotransferase (14.8%) were the most common AEs; in DMF-treated patients, AEs were 88.2% with predominance of flushing (73.2%), pruritus (16.9%), and abdominal pain (16.9%).

**Conclusion:**

Based on our findings, DMF is as efficacious and safe as TRF for the treatment of RRMS in our Iranian study population. Multicentric studies need to corroborate these findings in other populations.

## 1. Introduction

Over the past 20 years, the outcome of patients with multiple sclerosis (MS) has significantly improved with earlier diagnosis by magnetic resonance imaging (MRI) and earlier administration of disease-modifying therapies (DMTs) [1]. DMTs consist of treatments targeting the immune system to reduce autoimmune damage to the central nervous system and slow down the natural history of MS [2, 3].

Despite all of the advantages of DMTs, they may cause adverse events (AEs) with different severities leading to nonadherence, decreased quality of life, or treatment discontinuation [4–6]. This has been more problematic in platform agents like Interferons. An 8-year study of Interferon-Beta use in 394 cases with remitting-relapsing MS (RRMS) or secondary-progressive MS (SPMS) patients revealed 14% of treatment discontinuation due to AEs including flu-like syndrome, injection-site reaction, depression, and fatigue [7]. Another 5-year study on 122 Interferon-Beta-treated RRMS patients reported 23% treatment discontinuation due to either clinical AEs such as local injection-site reaction and flu-like syndrome or laboratory AEs including leukopenia and elevated liver enzymes [8].

Teriflunomide (TRF) and dimethyl fumarate (DMF) are approved oral DMTs that have proven efficacy in reducing annualized relapse rate, T2 lesion accrual, and short-term disability progression among RRMS patients compared with placebo [9–12]. Despite having the convenience of oral administration as opposed to platform injectable DMTs, AEs such as gastrointestinal irritability and elevated liver enzymes result in nonadherence or treatment discontinuation [10, 12, 13].

Awareness of efficacy, tolerability, and AEs of these agents is of significant importance to both treating physicians and patients [14, 15]. However, there is limited study in the literature assessing the adverse effects of these two oral agents. Consequently, we carried out this study to compare the efficacy and safety of TRF and DMF.

## 2. Methods

### 2.1. Study Population

The current observational study has been conducted on adults with remitting-relapsing multiple sclerosis diagnosed with relapsing-remitting MS, fulfilling the 2017 McDonald diagnostic criteria [16], who were referred to the outpatient Multiple Sclerosis Clinic of Kashani Hospital, affiliated with Isfahan University of Medical Sciences.

RRMS patients with age range of 18–65 years who started treatment with TRF or DMF between April 2015 and June 2017 (previously treated with platform first-line DMTs including Interferons or Glatiramer Acetate or treatment-naïve) with documented baseline expanded disability status scale (EDSS) within six months prior to treatment initiation and with the EDSS range of 0 to 5.5 at the treatment baseline were included. All subjects provided written consent to participate in the study.

Exclusion criteria included the following: presence of comorbid chronic illnesses confounding the assessment of efficacy or side effects, inability to adhere to follow up appointments, the patient's reluctance for participation in the study, concurrent use of chemotherapeutic, cytotoxic, or other agents causing similar adverse effects, and previous treatment with the second-line DMTs (including rituximab, natalizumab, cyclophosphamide, alemtuzumab, mitoxantrone, and fingolimod).

The TRF-treated patients were under treatment with the daily dose of 14 mg and the DMF-treated patients were under treatment with 240 mg twice daily after initial escalation period.

The study was approved by Ethics Committee of Isfahan University of Medical Sciences (REB fine number: IR.MUI.MED.REC.1398.386). Written consent for participation in the study was obtained from all subjects who were willing to participate.

### 2.2. Primary Outcome

The primary endpoint of our study was to compare the confirmed disability progression (CDP) following the use of TRF versus DMF. Disability progression was defined as an increase in the EDSS score of ≥1.0 point when the baseline EDSS score is equal to or more than 1 or an increase of ≥1.5 points when the baseline EDSS score is equal to zero. This increase was confirmed after 12 weeks.

### 2.3. Secondary Outcomes

The main secondary endpoints of this study included assessment of TRF and DMF efficacy and adverse effects divided into short-term and long-term AEs as follows:

1. TRF short-term AEs: nausea, diarrhea, headache, and rash
2. TRF long-term AEs: liver dysfunction, neutropenia, and leukopenia
3. DMF short-term AEs: flushing and gastrointestinal symptoms (e.g., diarrhea and abdominal pain)
4. DMF long-term AEs: lymphopenia, liver dysfunction, increased levels of bilirubin, and serum aminotransferases (alanine transferase and aspartate transferase)

Demographic and clinical data of the study population were extracted from our database [17]. The demographic information included age at disease onset, age at the last follow-up visit, duration of the disease, gender, educational level, and occupational status.

Clinical information included the first manifestation, previous history of DMTs therapy before TRF or DMF treatment initiation, baseline expanded disability status scale (EDSS) at the time of TRF or DMF initiation and last follow-up visit, number of relapses within 36 months prior to TRF or DMF initiation, number of relapses within 12 months prior to TRF or DMF initiation, number of patient-reported relapses during follow-up period, annualized relapse rate, confirmed disability improvement (CDI), and confirmed disability progression (CDP) within 12 weeks prior to the last follow-up visit. EDSS score was measured by treating neurologist [18].

**Figure 1:**
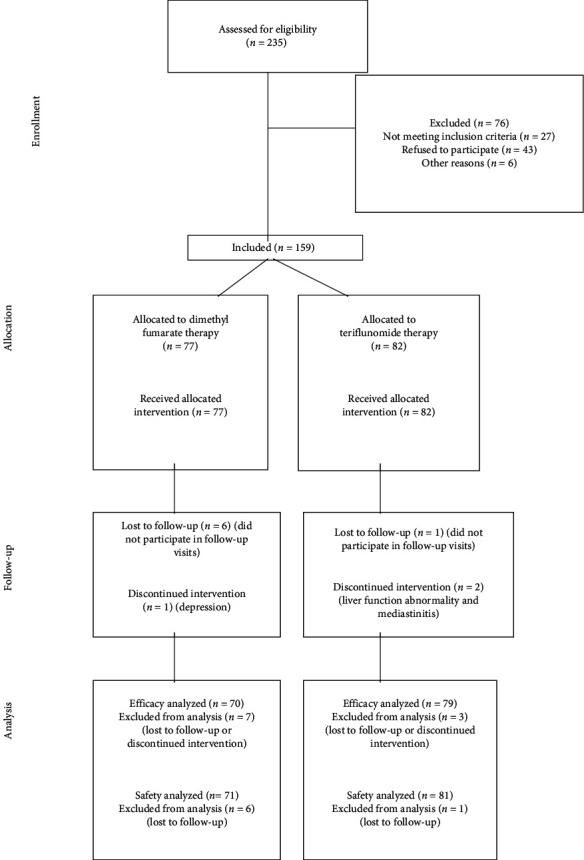
The study flowchart.

As this study data has been obtained from a real-time practice, there were no scheduled study visits. All the patients were assessed at the baseline and a follow-up visit. The baseline visit and EDSS were the assessments performed between 3 months before initiation and 6 months after initiation of the oral agent. In case of multiple visits during this period, the closest time to the initiation of TRF or DMF treatment was considered the baseline visit and EDSS. The follow-up visit and assessments were between 18 and 24 months following the initiation of the treatments. In cases of multiple visits, the closest visit to the second anniversary of TRF or DMF treatment was recorded. The study flowchart is shown in Figure 1.

The diagnosis of relapse was made by treating neurologist of the study as any new or worsening neurological symptoms compatible with relapses of MS lasting more than 24 hours in absence of fever, systemic illness, or significant psychological distress.

### 2.4. Statistical Analysis

Obtained data were entered into the Statistical Package for Social Sciences (SPSS) version 23. The descriptive data were presented in mean, standard deviation, percentages, and absolute numbers. The propensity score matching test was utilized to estimate the average efficacy of the treatments, using nearest-neighbor matching and a match tolerance of 0.1. The covariate variables in this analysis were as follows: age at MS diagnosis, current age, sex, duration of disease, history of previous immunosuppressive treatment, baseline EDSS, and number of relapses in prior year. The patients who were not included in the matching were excluded. In order to analyze the matched and unmatched indices, we conducted Student's *t*-test and Mann–Whitney *U* test to compare continues variables. Chi-square test was used to compare categorical variables. We used logistics regression analysis to compare outcomes between study groups. *P* value less than 0.05 was considered significant.

## 3. Results

The primary endpoint of the study was to assess the efficacy and safety of two oral DMTs in 159 RRMS patients (82 TRF-treated and 77 DMF-treated patients). In the study cohort, one patient in TRF group and six patients in DMF group withdrew from the study. Two patients in TRF group (one due to mediastinitis and one because of abnormally elevated live enzymes) and one patient in DMF group (due to depression) discontinued the treatment. Efficacy and safety evaluations were not done in subjects lost to follow-up, while the cases that altered the treatment due to AEs were included in safety assessments but not in efficacy assessments (the study results were not based on intention-to-treat groups). From 79 patients who received TRF, 32 patients were treatment-naïve and received it as a first-line agent, while, in the DMF group, 38 out of 79 ones received DMF as the first-line agent. All of the other patients with previous exposure to DMTs were on either Beta-Interferon or Glatiramer Acetate. Intolerance of platform agents (IF-beta or GA) was the reason for switch to TRF or DMF in 35.2% and 38.2% of the patients, respectively. Ineffectiveness of platform agents was the reason for switch in 16.1% of patients in the DMF-treated group and 15.3% of patients in the TRF-treated group.

### 3.1. Propensity Score Matching

Considering the observational study design, clinical and demographic data were matched using propensity score matching. The confounding variables in the current study included age of TRF vs. DMF treatment initiation onset, age at the last follow-up visit, gender distribution, duration of the disease, history of DMT administration before the index time of TRF vs. DMF initiation, baseline EDSS prior to TRF vs. DMF initiation, and numbers of relapses within 12 months prior to the TRF vs. DMF initiation.  Table 1 demonstrates the demographic and clinical information of the studied groups before and after matching.

### 3.2. Efficacy Evaluations

Propensity score matching was used to assess the efficacy of the regimens. As shown in Table 2, before the matching, the EDSS of the TRF-treated patients was higher than that of DMF group with statistical significance (*P* value = 0.027); the difference was not statistically significant after matching (*P* value = 0.542).

The 12-week CDP was statistically significantly higher among the TRF-treated patients compared to DMF group before matching. Following matching, 12-week CDI and 12-week CDP comparison of the two treatments revealed nonsignificant difference between the two therapeutic agents (*P* value ˃ 0.05).

As presented in Table 2, the number of patients who experienced relapses during the study (*P* value > 0.99) and the annualized relapse rate (ARR) (*P* value > 0.99) were not significantly different between the two groups following matching.

### 3.3. Safety Evaluations

Tables 3 and 4 summarize details of adverse events in DMF- and TRF-treated groups. In DMF-treated group, 88.2% had at least one adverse effect: flushing (73.2%), pruritus (16.9%), and abdominal pain (16.9%) were the most common ones (Table 2).

66 patients (81.4%) of the TRF-treated group reported one of the drug-related AEs. Hair thinning (hair loss) (62.9%), nail loss (20.9%), and liver function abnormality (14.8%) were the most common AEs associated with TRF.

## 4. Discussion

Comparison of the efficacy and safety of oral therapeutic agents for relapsing-remitting MS is difficult due to the lack of head-to-head randomized clinical trials. Lack of detailed comparative information about the advantages and disadvantages makes the choice of oral agents challenging for treating physician and patients. In the current study, we aimed to compare TRF with DMF in a real-life setting in a cohort of Iranian RRMS patients followed up for 2 years. The data were collected through a standardized method and analyzed using propensity score matching. The primary outcomes of this report included a comparison of CDP of the patients under treatment of either TRF or DMF. Clinical outcomes consisting of CDP, CDI, EDSS score, number of relapses, and number of patients with at least one relapse were not significantly different between the two agents following the matching of the two assessed groups. Two patients who received TRF and one on DMF withdrew from the study due to adverse events. The numbers of patients who experienced relapses during the year prior to the last visit and ARR were similar in two groups.

As far as we know, there are six studies comparing TRF treatment with DMF in multiple sclerosis patients. Hutchinson et al. did a network meta-analysis assessing the RCTs conducted to compare different molecules used for the treatment of multiple sclerosis [19]. The next two studies performed by Boster et al. [20] and Ontaneda et al. [14] were derived from the United States database; and the rest were observational studies based on databases in Italy [15], France [2], and Germany [21]. Three of these studies were indicative of superiority of DMF to TRF in terms of efficacy in the treatment of RRMS [14, 20, 21]. Two of the studies had similar outcomes to our data, as they found similar efficacy of both regimens for RRMS patients [2, 15].

We believe studies conducted in USA might have some biases due to lack of consideration of potential confounders in interpretation of results [14, 20]. Heterogeneity of the studies included in the meta-analysis by Hutchinson et al. might have resulted in difficulty in interpretation of outcomes related to efficacy of different agents [19]. The other studies had the advantages of including a large number of studied population, patient selection in real-life practice, and propensity matching for statistical analysis [2, 15, 21].

In our study, there were more AEs in DMF group compared to TRF group; treatment discontinuation occurred in one patient under DMF treatment due to depression and in two cases in TRF treatment due to abnormal liver function and mediastinitis. The proportion of AEs resulting in withdrawal was lower in our study compared with previous studies, which is probably the result of our small sample size. Most of the studies in the literature have reported higher rates of AEs in TRF than in DMF [15].

The most common AEs due to DMF were flushing, abdominal discomfort, and pruritus, which were similar to other reports. These AEs plus diarrhea (12.6% in our study) are the most common DMF-related AEs in the literature [15, 22].

In our study, there was only one case of treatment discontinuation in DMT due to depression, while in other reports in the literature there is around 12% of DMF discontinuation due to severe AEs including MS relapse, gastritis, gastroenteritis, pneumonia, leucopenia, ovarian cyst, and malignancy (12). On the other hand, a five-year interim analysis of ENDORSE trial reported the incidence of discontinuation due to severe AEs between 1 and 4% [23].

Hair thinning (hair loss) was the most common AE of TRF in our study, followed by nail loss and abnormal elevation of aminotransferase. Nail loss reported in 20.9% of our patients was an event that has not been commonly reported in the literature [24], while elevated blood pressure is a common TRF-associated AE (15) that was not experienced by our patients.

A 9-year follow-up study assessing the use of TRF for RRMS reported treatment discontinuation in 11% of the patients due to AEs and the incidence of severe AEs in up to 20% of TRF-treated cases [25].

Our study has some limitations. A significant limitation of our study is lack of information about baseline MRI findings. Short duration of follow-up is another limitation of the study. It is possible that because of this we could not find difference between the DMTs. The exact times of relapse in all patients were not documented. Therefore, we were unable to conduct a survival analysis. Another potential limitation (not only in our study but perhaps also in other studies) is presence of unrecognized confounder factors to be included in propensity score matching. For instance, factors such as smoking, patients' quality of life, sense of health, or mood and affect have not been collected in our database [26, 27]. On the other hand, selection bias may occur in real-life practice as the chance of having regular follow-up visits is higher in patients with active disease compared to patients with more quiescent disease [28]. Future studies with larger study population addressing issues related to selection bias and more extensive coverage of confounding factors will be of extreme value in providing more accurate conclusions.

In summary, we performed a propensity matching study on RRMS patients to compare the efficacy and safety of TRF versus DMF, in which the confounder factors were determined as age at the time of treatment initiation with TRF or DMF, age at the last follow-up visit, gender, duration of the disease, history of DMT exposure prior to initiation of TRF or DMF, baseline EDSS prior to initiation of TRF or DMF, and numbers of relapses within 12 months prior to initiation of DMF or TRF. The findings of our study revealed similar outcomes in terms of EDSS, CDI, CDP, and ARR between the two agents. The AEs that led to drug discontinuation were limited to a patient in the DMF-treated group due to depression and two patients in the TRF-treated group due to liver dysfunction and mediastinitis. AEs occurred in 88.2% of DMF-treated patients, among which flushing, pruritus, and abdominal pain were the most common; meanwhile AEs occurred in 81.4% of the TRF group with the predominance of hair loss, nail loss, and elevated aminotransferase. Based on our two-year study, DMF and TRF have similar efficacy for the treatment of RRMS in our cohort of Iranian patients.

## Figures and Tables

**Table 1:** Demographic and clinical information of the studied groups before and after matching.

Variable		All patients			Matched group		
		DMF (*N* = 70)	TRF (*N* = 79)	Statistics	DMF (*N* = 38)	TRF (*N* = 38)	Statistics
Age at disease onset, *y*, mean (SD)		25.17 ± 7.23	32.28 ± 10.06	MD = 7.10, *t* (145) = 4.859, *P* < 0.001	27.52 ± 6.96	28.56 ± 9.11	MD = 1.04, *t* (67.41) = 0.555, *P* = 0.581
Age at the last visit, *y*, mean (SD)		29.47 ± 8.18	40.72 ± 10.21	MD = 11.25, *t* (147) = 7.354, *P* < 0.001	33.15 ± 7.74	34.92 ± 9.18	MD = 1.76, *t* (74) = 0.905, *P* = 0.369
Female, *n* (%)		57 (81.4%)	68 (86.1%)	*X* ^2^ (*N* = 149, df = 1) = 0.593, *P* = 0.441	32 (84.2%)	33 (86.8%)	*X* ^**2**^ (*N* = 76, df = 1) = 0.106, *P* = 0.744
Education level, *n* (%)	Nonacademic	25 (35.7%)	47 (59.5%)	*X* ^2^ (*N* = 149, df = 1) = 8.404, *P* = 0.004	22 (57.9%)	22 (57.9%)	*X* ^2^ (*N* = 76, df = 1) = 7.773, *P* = 0.005
	Academic	45 (64.3%)	32 (40.5%)		16 (42.1%)	16 (42.1%)	
Employment status, *n* (%)	Employment	26 (37.1)	26 (37.1%)	*X* ^2^ (*N* = 149, df = 1) = 0.177, *P* = 0.674	14 (36.8%)	15 (39.5%)	*X* ^2^ (*N* = 76, df = 1) = 0.056, *P* = 0.813
	Unemployment	44 (62.9%)	32 (40.5%)		24 (63.2%)	23 (60.5%)	
First manifestation, *n* (%)	Visual	18 (25.7%)	28 (35.4%)	*X* ^2^ (*N* = 149, df = 1) = 2.435, *P* = 0.296	13 (34.2%)	13 (34.2%)	*X* ^2^ (*N* = 70, df = 1) = 0.500, *P* = 0.779
	Motor	29 (41.4%)	37 (46.8%)		18 (47.2%)	19 (50.0%)	
	Brain and brainstem	13 (18.6%)	9 (11.4%)		6 (15.8%)	6 (15.8%)	
DMT treatment prior to the TRF/DMF treatment initiation, *n* (%)		32 (45.7%)	47 (59.5%)	*X* ^2^ (*N* = 149, df = 1) = 2.829, *P* = 0.093	24 (63.2%)	24 (63.2%)	*X* ^2^ (*N* = 76, df = 1) = 0.000, *P* = 1.000
Disease duration, *y*, median (IQR)		3.5 (1.0–6.0)	6.0 (2.0–12.0)	*U* = 1719, *P* < 0.001	5.0 (1.7–8.2)	5.0 (2.0–10.2)	*U* = 705.5, *P* = 0.863
EDSS prior to the TRF/DMF treatment initiation, median (IQR)		1.5 (0.0–2.0)	2.0 (1.0–2.0)	*U* = 2555.5, *P* = 0.403	1.25 (0.0–2.0)	1.5 (0.0–2.0)	*U* = 697.0, *P* = 0.787
Relapses within 36 months prior to the TRF/DMF treatment initiation, mean (SD)		0.71 ± 0.54	0.56 ± 0.52	MD = −0.14, *t* (147) = −1.656,*P* = 0.100	0.65 ± 0.58	0.63 ± 0.54	MD = -0.02, *t* (74) = -0.204, *P* = 0.839
Patients with at least one relapse within 36 months prior to the TRF/DMF treatment initiation, *n* (%)		47 (67.2%)	44 (55.7%)	*X* ^2^ (*N* = 149, df = 1) = 2.045, *P* = 0.153	23 (60.6%)	23 (60.6%)	*X* ^2^ (*N* = 76, df = 1) = 0.000, *P* = 1.000
Relapses within 12 months prior to the TRF/DMF treatment initiation, mean (SD)		0.31 ± 0.46	0.15 ± 0.39	MD = −0.16, *t* (147) = −2.297, *P* = 0.025	0.26 ± 0.44	0.23 ± 0.48	MD = -0.02, *t* (74) = -0.245, *P* = 0.807
Patients with at least one relapse within 12 months prior to the TRF/DMF treatment initiation, *n* (%)		22 (31.4%)	11 (14.1)	*X* ^2^ (*N* = 149, df = 1) = 6.595, *P* = 0.010	10 (26.3%)	8 (21.1%)	*X* ^2^ (*N* = 76, df = 1) = 0.291, *P* = 0.589

DMF: dimethyl fumarate; TRF: teriflunomide; DMT: disease-modifying therapy; EDSS: expanded disability status scale; MD: mean difference.

**Table 2:** Comparison of outcomes between groups before and after propensity score matching.

Outcomes	All patients				Matched group			
	DMF (*N* = 70)	TRF (*N* = 79)	OR (95% CI)	*P* value	DMF (*N* = 38)	TRF (*N* = 38)	*P* value	*P* value
EDSS at the last visit, median (IQR)	0.0 (0.0–1.25)	1.0 (0.0–2.0)	0.692 (0.519, 0.922)	0.012	0.0 (0.0–1.5)	0.0 (0.0–2.0)	0.867 (0.546, 1.664)	0.867
Patients with 12-week CDP, *n* (%)	3 (4.3%)	15 (19.0%)	0.191 (0.053, 0.691)	0.012	2 (5.3%)	4 (10.5%)	0.479 (0.058, 3.969)	0.495
Patients with 12-week CDI, *n* (%)	24 (34.3%)	26 (32.9%)	1.064 (0.538, 2.101)	0.859	11 (28.9%)	12 (31.6)	0.805 (0.293, 2.213)	0.675
Patients with relapse during follow-up, *n* (%)	3 (4.3%)	3 (3.8%)	1.134 (0.221, 5.811)	0.880	2 (5.3%)	2 (5.3%)	1.146 (0.129, 10.202)	0.903

DMF: dimethyl fumarate; TRF: teriflunomide; EDSS: expanded disability status scale; CDP: confirmed disability progression; CDI: confirmed disability improvement.

**Table 3:** Adverse events reported in all patients treated with dimethyl fumarate.

	Dimethyl fumarate, *N* = 71
Patients with at least one adverse event, *n* (%)	63 (88.2%)

The proportion of patients with an adverse event, *n* (%)	
Flushing	52 (73.2%)
Pruritus	12 (16.9%)
Abdominal pain	12 (16.9%)
Liver dysfunctional test	11 (15.4%)
Nausea	11 (15.4%)
Dry mouth	9 (12.6%)
Diarrhea	9 (12.6%)
Itching	8 (11.2%)
Weight loss	7 (9.8%)
Dyspnea	3 (4.2%)
Palpitation	1 (1.4%)
Tremor	1 (1.4%)
Hair loss	1 (1.4%)
Depression	1 (1.3%)

**Table 4:** Adverse events reported in all patients treated with teriflunomide.

	Teriflunomide, *N* = 81
Patients with at least one adverse event, *n* (%)	66 (81.4%)

The proportion of patients with an adverse event, *n* (%)	
Hair thinning (hair loss)	51 (62.9%)
Nail loss	17 (20.9%)
Liver dysfunctional test	12 (14.8%)
Itching	8 (9.8%)
Nausea	8 (9.8%)
Pruritus	5 (6.1%)
Dyspnea	4 (4.9%)
Diarrhea	3 (3.7%)
Paresthesia	3 (3.7%)
Headache	2 (2.5%)
Psychiatric disorder	2 (2.5%)
Mediastinitis	1 (1.2%)
Flushing	1 (1.2%)
Abdominal pain	1 (1.2%)
Urinary tract infection	1 (1.2%)
Recurrent urinary tract infection	1 (1.2%)
Dry mouth	1 (1.2%)
Eye disorder	1 (1.2%)

## Data Availability

The data used to support the findings of this study are available from the corresponding author upon request.
